# Importance of Adequate Surgical Local Control in Fusion-Negative Para-Testicular Rhabdomyosarcoma: Data From the Cooperative Weichteilsarkom Studiengruppe Trials (CWS-96 and CWS-2002P) and the European Soft Tissue Sarcoma Registry (SoTiSaR)

**DOI:** 10.1245/s10434-024-15568-3

**Published:** 2024-06-15

**Authors:** Illya Martynov, Monika Sparber-Sauer, Amadeus Heinz, M. Christian Vokuhl, Martin Ebinger, Jens Gesche, Marc Münter, Ewa Koscielniak, Jörg Fuchs, Guido Seitz, Cooperative Soft Tissue Sarcoma Study Group

**Affiliations:** 1 Department of Pediatric Surgery and Urology, University Hospital Giessen-Marburg, Philipps-University, Campus Marburg, Marburg, Germany; 2 Department of Pediatric Surgery, University Hospital Giessen-Marburg, Campus Giessen, Giessen, Germany; 3 Pädiatrie 5 (Pädiatrische Onkologie, Hämatologie, Immunologie), Klinikum der Landeshauptstadt Stuttgart gKAöR, Olgahospital, Stuttgart Cancer Center, Zentrum für Kinder-, Jugend- und Frauenmedizin, Stuttgart, Germany; 4 University of Medicine Tübingen, Tübingen, Germany; 5 Department of Pediatric Hematology and Oncology, University Children´s Hospital, Tuebingen, Germany; 6 Section of Pediatric Pathology, Department of Pathology, University Hospital Bonn, Bonn, Germany; 7 Pediatric Surgery, Josefinum, Augsburg, Germany; 8 Department of Radiooncology, Klinikum Stuttgart, Stuttgart, Germany; 9 Department of Pediatric Surgery and Urology, University Children’s Hospital, Tuebingen, Germany

**Keywords:** Paratesticular rhabdomyosarcoma, Surgery, Protocol violations, Pretreatment re-excision, Retroperitoneal lymph node dissection

## Abstract

**Background:**

This study aimed to assess the impact that the quality of primary and subsequent surgeries has on the survival of patients with para-testicular rhabdomyosarcoma (PTRMS).

**Methods:**

Patients with localized (IRS I–III) and metastatic (IRS IV) PTRMS were enrolled in the two Cooperative Weichteilsarkom Studiengruppe (CWS) trials (CWS-96, CWS-2002P) and the Soft Tissue Sarcoma Registry (SoTiSaR).

**Results:**

Among 196 patients (median age, 8.4 years), 106 (54.1%) had primary complete resection. Image-defined lymph node (LN) disease was detected in 21 (11.5%) patients in the localized cohort and 12 (92.3%) patients in the metastatic cohort. The 5-year event-free survival (EFS) and overall survival (OS) were respectively 87.3% and 94.0% for the patients with localized PTRMS and 46.2% and 42.2% for the patients with metastatic PTRMS. Protocol violations during the primary surgery (PV-PS) were observed in 70 (42%) of the IRS I–III patients. This resulted in higher rates of R1/R2 resections (*n* = 53 [76%] vs *n* = 20 [21%]; *p <* 0.001) with a need for pretreatment re-excision (PRE) (*n* = 50 [83%] vs *n* = 10 [17%]; *p* < 0.001) compared with the patients undergoing correct primary surgery. Protocol violations during PRE occurred for 13 (20%) patients. Although PV-PS did not influence the 5-year EFS or OS in the localized PTRMS cohort, the unadjusted log-rank test showed that R status after PRE is a prognostic factor for 5-year OS (R1 vs R0 [81.8% vs 97.6%]; *p* = 0.02).

**Conclusions:**

The quality of surgical local control in PTRMS is unsatisfactory. Emphasis should be placed on evaluating the resection status after PRE in further clinical trials.

**Supplementary Information:**

The online version contains supplementary material available at 10.1245/s10434-024-15568-3.

Para-testicular rhabdomyosarcoma (PTRMS) is the most prevalent non-germ-cell scrotal malignant tumor, accounting for approximately 5% of all pediatric scrotal masses and 7% of all pediatric rhabdomyosarcomas (RMSs).^1–3^ Patients with PTRMS have their condition diagnosed mostly at a median age of 5–8 years. They usually present initially with a painless, palpable, solid scrotal mass.^4–6^

Due to the likelihood of early clinical detection and the predominance of the favorable fusion-negative embryonal histologic subtype, the prognosis of localized PTRMS is excellent, with a reported 5-year overall survival (OS) of approximately 95%.^7^ However, up to 25% of patients with PTRMS are prone to have histologically confirmed retroperitoneal lymph node (LN) disease at presentation,^8^ and approximately 5% have distant metastases, with a reported 3-year event-free survival (EFS) of 40%.^9^ Patient age (≥ 10 years) and tumor size (> 5 cm) are consistently identified as negative prognostic factors affecting both EFS and OS in PTRMS, attributable to a higher prevalence of positive retroperitoneal LNs and increased rates of microscopic margin status.^7^

Primary inguinal orchidectomy with high ligation of the spermatic cord before tumor mobilization is the recommended surgical approach for PTRMS. For cases in which the tumor has invaded the scrotal skin, hemiscrotectomy is recommended.^10^ Tumor resection status is a major prognostic factor, with reported 5-year EFS and OS rates of respectively 88% and 96% for International Rhabdomyosarcoma Study (IRS) group I (complete tumor resection, R0), 89% and 93% for IRS group II (gross tumor resection with microscopically positive margins, R1) compared with 61% and 68% for IRS group III (gross residual disease after resection, R2) patients.^7^ For cases of incomplete tumor resection with preservation of the testicle or inappropriate inguinal or trans-scrotal surgery carrying the risk of tumor spillage and contamination, pretreatment re-excision (PRE) before initiation of adjuvant chemotherapy is recommended.^10^ Because PRE leads to downstaging from IRS III or IRS II to IRS I, excellent EFS and OS outcomes can be achieved.^11^ However, available data on the impact of the failed PRE are sparse, leading to R1/R2 status on EFS or OS for patients with PTRMS.

This study aimed to assess the impact that quality of the primary and subsequent surgeries had on the survival of patients with non-metastatic (IRS I–III) and metastatic (IRS IV) PTRMS enrolled in two prospective Cooperative Weichteilsarkom Studiengruppe (CWS) trials (CWS-96, CWS-2002P) and one European Soft Tissue Sarcoma Registry (SoTiSaR). Furthermore, we examined the diagnostic relevance of surgical LN staging by analyzing its association with the prevalence of radiologically normal and abnormal LNs.

## Methods

### Patients

Patients with reference histologically confirmed RMS were prospectively enrolled and treated according to the related protocols of two CWS trials (CWS-96, CWS-2002P) and CWS Guidance.^12–14^ European patients with PTRMS not treated in the RMS 2005 study were registered in the European Soft Tissue Sarcoma Registry (SoTiSaR) since 2009 and treated according to CWS Guidance. The CWS Guidance reflects the standard regimen of the RMS 2005 study.^15,16^ The institutional review board of the Landesarztekammer Baden-Württemberg granted approval for CWS-96 (105/95), and the University of Tübingen provided approval for CWS-2002P (51/2003) and SoTiSaR (158/2009B02). Written informed consent for participation was obtained from the patients and/or parents before enrollment.

Of the initial 241 patients with PTRMS enrolled in CWS-96 (*n* = 91), CWS-2002P (*n* = 54), and SoTiSar (*n* = 71), 45 were excluded due to an age older than 21 years, incomplete data, withdrawal of informed consent, or the histologically fusion-positive alveolar RMS subtype. This resulted in a final cohort of 196 patients (CWS-96 [*n* = 71], CWS-2002P [*n* = 54], SoTiSaR [*n* = 71]). Among these 196 patients, 183 had localized (IRS I–III) and 13 had metastatic (IRS IV) PTRMS.

### Risk Stratification and Treatment

The patients were stratified according to the risk stratification RMS/CWS-96^12^ and common CWS/EpSSG stratification according to CWS-2002P^13^ and CWS Guidance/RMS 2005^17^ to low-risk (LR), standard-risk (SR), high-risk (HR) and very-high-risk (VHR) groups based on N status, histology, IRS group, and tumor site and size/patient’s age.^15^ The following LN sites were considered as non-metastatic regional: the ipsilateral retroperitoneal LN up to the level of the renal pedicle (spermatic cord), the external iliac and ipsilateral pelvic LN (epididymis), and the inguinal LN (scrotal skin). Lymph node involvement above the renal vessels or at other sites was considered as distant metastasis (“metastatic LN”).

Magnetic resonance imaging (MRI) with the administration of contrast agent using conventional sequences (T1, T2, post-contrast) in combination with diffusion-weighted imaging (DWI) sequences was recommended for staging and primary assessment of both the tumor and LNs, whereas computed tomography (CT) scan was specifically suggested for lung evaluation. The criteria used to identify radiologically pathologic LNs involved a short-axis measurement of 10 mm or greater. Treatment of the patients consisted of polychemotherapy based on their risk stratification and the specific trial protocol and radiotherapy (RT) if indicated, as presented in Table S1.

### Definitions, Local Surgery, and LN Surgery

The surgical protocol consisted of a high radical inguinal orchiectomy with the clamping of the spermatic cord at the level of the internal inguinal ring before tumor mobilization. This surgical approach was not considered mutilating. Histologic workup of resection margins defined the postsurgical staging according to the IRS groups, with IRS I defined as microscopically complete tumor resection (R0), IRS II defined as macroscopically complete resection (R1), and IRS III defined as macroscopically incomplete tumor resection (R2).^18^

Protocol violations (PVs) during primary surgery were defined as any deviations from surgical guidelines for PTRMS resection, including trans-scrotal access, testicular-sparing surgery, and no clamping of the spermatic cord during tumor resection. Furthermore, the placement of a drain and avoidable intraoperative tumor spillage (e.g., deliberately opening the tumor capsule for macroscopic inspection of the tumor surface) also were classified as inappropriate surgery due to the high risk for tumor cell contamination of the operating field.

A hemiscrotectomy was indicated if the tumor invaded into the scrotal skin. Pretreatment re-excision was warranted for patients with microscopic residual disease or for cases in which PVs occurred, as recommended by the CWS study board.

According to the CWS study protocols and the CWS Guidance, primary surgical sampling of retroperitoneal LN was not recommended, even in the case of radiologically pathologic LN on initial imaging. If LNs were radiologically pathologic (>10 mm) after chemotherapy at the first reassessment, irradiation was deemed as effective as lymphadenectomy and, in the absence of contraindications, was the preferred option over lymphadenectomy.

### Statistical Analysis

The primary outcomes of the study were 5-year EFS and OS, calculated with Kaplan-Meier estimates and compared with the unadjusted log-rank test between survival curves. Because we were not able to perform a Cox regression analysis due to the low number of events per variable and the low prevalence of predictors,^19^ we additionally examined potential confounding factors using binomial uni- and multivariable logistic regression models. This allowed for a deeper consideration of how various factors may be influencing survival.

Overall survival was calculated as the time from the primary diagnosis to either therapy-related or non-therapy-related death or the last follow-up evaluation, which were considered censored observations. Event-free survival was calculated as the time from the initial diagnosis to the first event, defined as a disease relapse (including local, nodal, metastatic, or combined).

Categorical variables were described using frequencies and percentages. Fisher’s exact test was used for analysis of categorical variables. Continuous variables were reported as median with corresponding interquartile range (IQR). All statistical tests were two-tailed, and the level of significance was determined as *p* value lower than 0.05. Statistical analyses were conducted using IBM SPSS 26 (SPSS, Armonk, NY, USA).

## Results

### Patients

The median age of the patients in the IRS I–III cohort was 7.62 years (IQR, 4.8–15.6 years) and showed a bimodal distribution, with the first peak at 5 years and the second peak at 18 years of age. All the patients in the IRS IV cohort were older than 10 years, with a median age of 16.4 years (IQR, 14.8–17.7 years; Fig. S1). In the IRS I–III cohort, 42.1% of the patients were older than 10 years.

A primary tumor larger than 5 cm was reported for 40.4% of the patients in the IRS I–III cohort and for 84.6% of the patients in the IRS IV cohort. The primary surgery was performed in 71.7% of that cases at general hospitals and in 28.3% of the cases at university hospitals. The PTRMS patients had surgery by surgeons of various surgical disciplines including urologists (64.8%), pediatric surgeons (34.1%), and general surgeons (1.1%). Baseline characteristics by IRS stage are provided in Table 1.

**Table 1:** Demographic characteristics of patients by IRS stage

	IRS I–III (*n* = 183) *n* (%)	IRS IV (*n* = 13) *n* (%)
Cooperative group		
CWS-96	68 (37.2)	3 (23.1)
2002P	49 (26.8)	5 (38.5)
SoTiSaR	66 (36.1)	5 (38.5)
Age at enrollment (years)		
≤ 10	106 (57.9)	0 (0.0)
> 10	77 (42.1)	13 (100)
Size of primary tumor (cm)		
≤ 5	106 (57.9)	2 (15.4)
> 5	74 (40.4)	11 (84.6)
Unknown	3 (1.6)	0 (0.0)
Side distribution		
Left	94 (51.4)	5 (41.7)
Right	88 (48.1)	7 (58.3)
Bilateral	1 (0.5)	1 (7.7)
T stage		
T1	117 (63.9)	0 (0.0)
T2	51 (27.9)	11 (84.6)
TX	15 (8.2)	2 (15.4)
N stage (imaging)		
N0	139 (76.0)	1 (7.7)
N1	21 (11.5)	12 (92.3)
NX	23 (12.6)	0 (0.0)
T stage post-surgery		
pT0	1 (0.5)	0 (0.0)
pT1	107 (58.5)	0 (0.0)
pT2	30 (16.4)	1 (7.7)
pT3	2 (1.1)	0 (0.0)
pT3a	31 (16.9)	2 (15.4)
pT3b	8 (4.4)	9 (69.2)
pTX	4 (2.2)	1 (7.7)
Regional lymph node status (histology)		
pN0	32 (17.5)	0 (0.0)
pN1	8 (4.4)	3 (23.1)
pN1b	0 (0.0)	1 (7.7)
pNX	143 (78.1)	9 (69.2)
Histology		
Embryonal	154 (84.2)	11 (84.6)
Spindle cell	23 (12.6)	0 (0.0)
Alveolar (fusion-negative)	5 (2.7)	2 (15.4)
Unknown	1 (0.5)	0 (0.0)
Median follow-up: years (range)	6.02 (0.15–18.3)	1.67 (0.63–9.91)

*IRS* International Rhabdomyosarcoma Study Group stage; *CWS* Cooperative Weichteilsarkom Studiengruppe; *SoTiSaR* European Soft Tissue Sarcoma Registry

### Primary Surgery, Protocol Violations, and PRE

Primary surgery was either resection (93.4%) or biopsy (6.6%). In the IRS I–III cohort, the primary surgery methods included orchiectomy (66.1%), testicular-sparing tumor resection (29.5%), and primary hemiscrotectomy (1.1%). Data on the primary surgical approach were missing in 3.3% of the cases. In the localized PTRMS cohort, an R0 resection after the primary surgery was achieved in only 55.7% of the cases.

Protocol violations during primary surgery (PV-PS) were noted in 41.9% of the patients, including both single and multiple protocol-deviating events per patient. Trans-scrotal tumor resection with or without orchiectomy was the most prevalent (51.7%), followed by tumor mobilization without clamping of the spermatic cord (25.2%), drain usage (12.6%), and avoidable tumor spillage (10.5%).

The proportion of R1/R2 resections was significantly higher in the PV-PS cohort than among the patients who underwent an appropriate primary surgery (*n* = 53 [76%] vs *n* = 20 [21%]; *p <* 0.001).

Neither the patient’s age (≤10 vs >10 years) nor tumor size (≤5 vs >5 cm) increased the chance of protocol-deviating surgery or R1 status after primary resection (Table 2). Overall, PRE was required in 35.5% of the cases, with a significantly higher proportion among the patients with PV-PS than among those who underwent an appropriate primary surgery (*n* = 50 [83%] vs *n* = 10 [17%]; *p* < 0.001).

**Table 2:** Primary and secondary testicular surgery in IRS I–III study cohort by age and tumor size

	Age				Tumor size			
	Total	≤ 10 Years *n* (%)	> 10 Years *n* (%)	*p* Value	Total	≤5 cm *n* (%)	> 5 cm *n* (%)	*p* Value
Primary surgical approach								
Orchiectomy	121	70 (57.9)	51 (42.1)	0.395	120	68 (56.7)	52 (43.3)	0.703
Hemiscrotectomy with en bloc orchiectomy	2	2 (100)	0 (0.0)		2	1 (50.0)	1 (50.0)	
Testicular-sparing surgery	54	32 (59.3)	22 (40.7)		52	34 (65.4)	18 (34.6)	
Unknown	6	2 (33.3)	4 (66.7)		6	3 (50.0)	3 (50.0)	
Protocol violation during initial surgery								
Yes	70	40 (57.1)	40 (42.9)	0.522	68	39 (57.4)	29 (42.6)	0.629
No	97	61 (62.9)	36 (37.1)		97	60 (61.9)	37 (38.1)	
R status after primary surgery								
R0	102	59 (57.8)	43 (42.2)	0.55	102	62 (60.8)	40 (39.2)	0.647
R1	64	34 (53.1)	30 (46.9)	0.35	63	36 (57.1)	27 (42.9)	0.753
R2	19	14 (73.7)	5 (26.3)	0.219	17	10 (58.8)	7 (41.2)	0.595
R status after PRE								
R0	60	35 (58.3)	25 (41.7)	0.508	58	36 (62.1)	22 (37.9)	0.009
R1	9	7 (77.7)	2 (22.3)		11	2 (18.2)	9 (81.8)	
Surgical lymph node staging								
None	143	82 (57.3)	61 (42.7)	0.925	141	88 (62.4)	53 (37.6)	0.166
Inguinal	21	13 (61.9)	8 (38.1)		20	10 (50.0)	10 (50.0)	
Retroperitoneal	19	11 (57.9)	8 (42.1)		19	8 (42.1)	11 (57.9)	
Retroperitoneal lymph node involvement^a^								
Yes	8	1 (12.5)	7 (87.5)	0.001	8	1 (12.5)	7 (87.5)	0.059
No	11	10 (90.9)	1 (9.1)		11	7 (63.6)	4 (36.4)	

*IRS* International Rhabdomyosarcoma Study Group stage; *PRE* pretreatment re-excision

^a^Deviation from the number results from the missing variables of age or tumor size.

Remarkably, PVs during PRE were noted in 20% of all the patients. These PVs included drain usage (61.5%), tumor mobilization without clamping of the spermatic cord (15.4%), intentional high preperitoneal exploration with consecutive peritoneal breaching leading to tumor spillage into the peritoneal cavity (7.7%), trans-scrotal tumor resection (7.7%), and revisional inguinal orchiectomy instead of the recommended hemiscrotectomy due to orchidopexy (risk of potential tumor cell contamination of the scrotal skin) after para-testicular tumor resection during primary surgery (7.7%).

No differences regarding R status (R0 *vs*. R1) were found between PRE with PV and PRE without PV (*p =* 0.725). In 53.1% of the cases, PRE was performed at an institution other than the one performing the primary surgery. Interestingly, the incidence of PV during PRE was significantly higher among the patients whose PRE was performed at the same institution as the primary surgery than among the patients transferred to another institution (76.9% vs 23.1%; *p =* 0.01). Moreover, the chance of PV during PRE was higher if the PRE was performed in a non-university hospital rather than a university hospital (84.6% vs 15.4%; *p* < 0.001).

Revisional surgery after PRE (ReDo PRE) was performed for 4.5% of the patients. Final R0 status after PRE or ReDo PRE was achieved in 87.9% of the cases. Overall, in the IRS I–III cohort, R0 status after primary and/or revisional surgeries was not achieved for 8.7% of the patients. Figure S2 provides a detailed overview of the primary and revisional surgeries with the corresponding R status in the IRS I–III study cohort.

### LN Assessment and Treatment

In the IRS I–III cohort, 40 patients underwent surgical LN exploration, which included sampling of inguinal LNs (52.5%) and/or retroperitoneal LNs (47.5%). Of these LNs, 20% (*n* = 8) were categorized as pN1 and located exclusively in the retroperitoneal region. Considering only the retroperitoneal LNs sampled (*n* = 19, 47.5%), the proportion of pN1 LNs was 42.1% (8/19).

All affected retroperitoneal LNs were radiologically pathologic on initial imaging. In contrast, all inguinal LNs and retroperitoneal LNs without pathologic imaging findings were histologically tumor-free after surgical LN sampling (Fig. S3). Altogether, nine patients with pN1 and/or N1 received RT, and one patient received a combination of both RT and retroperitoneal LN dissection (RPLND). In the patients with metastatic PTRMS (*n* = 13), the regional RPLNs were radiologically pathologic in 92.3% and distant LN metastases in 61.5% of the cases, together with pulmonary metastases (*n* = 6, 46.1%) and skeletal metastases (*n* = 10, 76.9%). Combined metastases (LN, skeletal, lung) were detected in 12 (92.3%) patients.

### Outcome Measures

Among the IRS I–III patients, the 5-year EFS was 87.3% (95% confidence interval [CI], 82.1– 2.9%), and the 5-year OS was 94.0% (95% CI, 90.0–98.1%). In the IRS I–III patient cohort, 28 events were reported, including 6.6% metastatic recurrences (*n* = 12: 33% in children age ≤10 years and 67% in children age >10 years), 5.5% LN relapses (*n* = 10: 40% in children age ≤10 years and 60% in children age >10 years), and 3.3% local recurrences (n = 6: 50% in children age ≤10 years and 50% in children age >10 years).

Among the patients with metastatic PTRMS, the 5-year EFS was 46.2% (95% CI, 25.7–83.0%) and the 5-year OS was 42.2% (95% CI, 21.3–83.6%). In the IRS IV cohort, a total of seven events were reported, with 57.2% of these events being disease progression and 42.8% being combined disease recurrences.

**Fig. 1:**
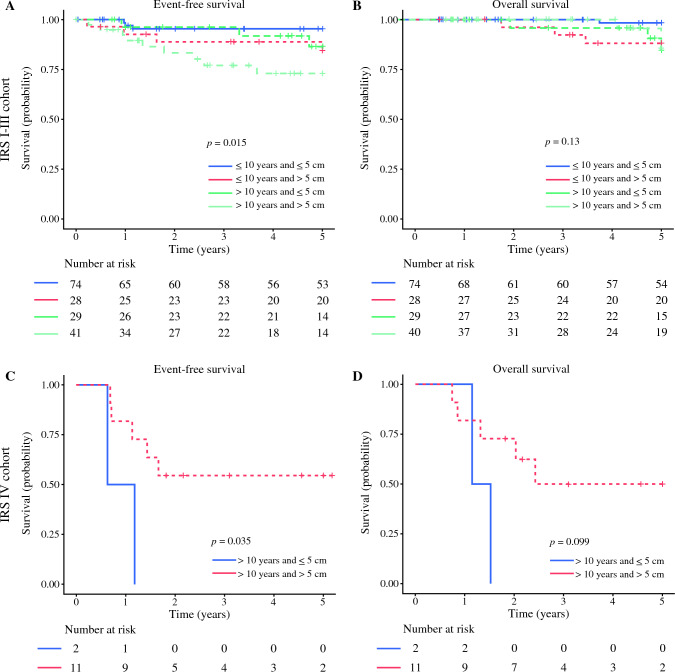
5-year EFS and OS analysis in IRS I–III and IRS IV cohorts by patient age and tumor size. EFS, event-free survival; OS, overall survival; IRS, International Rhabdomyosarcoma Study Group stage

**Fig. 2:**
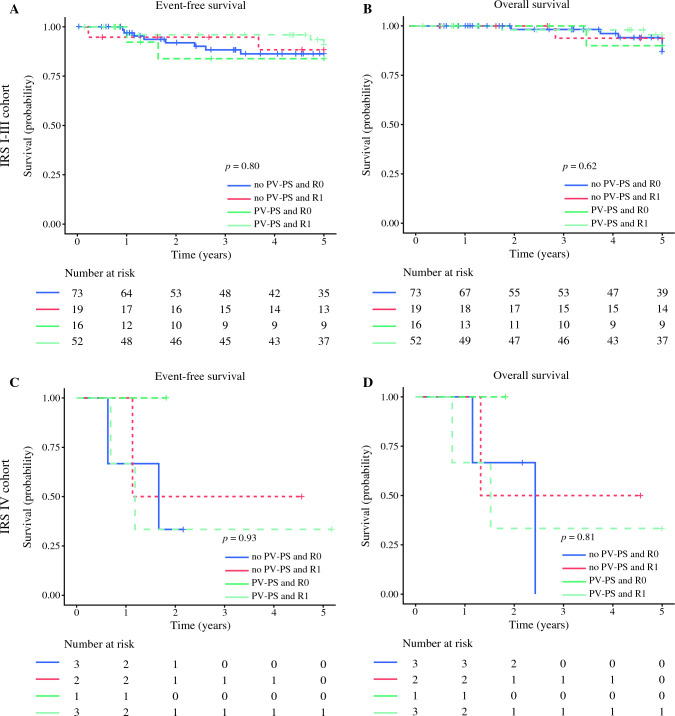
5-Year EFS and OS analysis in IRS I–III and IRS IV cohorts by surgery. EFS, event-free survival; OS, overall survival; IRS, International Rhabdomyosarcoma Study Group stage; PV-PS, protocol violation during primary surgery

In the IRS I–III cohort, EFS was significantly influenced by clinic-demographic factors, including patient age (*p =* 0.01), tumor size (*p =* 0.006), and presence of bilateral tumors (*p <* 0.0001), whereas OS was significantly affected only by bilateral tumors (*p <* 0.001) (Table 3). By combining the data for patient´s age and tumor size in the IRS I–III cohort, we found that the patients older than 10 years with tumor larger than 5 cm had the most unfavorable EFS (*p =* 0.01), whereas OS was not affected (*p =* 0.13) (Fig. 1). Analysis of combined surgical variables (PV [yes/no] and R0 status [yes/no]) showed no significant differences in the EFS and OS rates (EFS [*p =* 0.80]; OS [*p =* 0.62]; Fig. 2).

**Table 3:** 5-Year OS and EFS analysis in localized (IRS I–III) PTRMS

Variable	IRS I–III			
	5-Year OS % (95% CI)	*p* Value	5-Year EFS % (95% CI)	*p* Value
Cooperative group: *n* (IQR)				
CWS-96 vs CWS-2002P vs SoTiSaR	93.5 (87.6–99.9) vs 93.3 (86.3–100) vs 88.2 (76.3–100)	0.44	85.8 (77.6–94.9) vs 91.5 (83.8–99.8) vs 83.8 (73.4–95.7)	0.33
Age: years (IQR)				
≤10 vs >10	95.2 (90.8–99.9) vs 85.9 (75.9–97.2)	0.12	92.3 (87.0– 8.0) vs 76.8 (66.1–89.1)	0.01
Tumor size: cm (IQR)				
≤5 vs >5	94.9 (90.1–99.9) vs 87.5 (78.5–97.5)	0.13	93.2 (88.0–98.6) vs 77.9 (67.8–89.5)	0.006
Side distribution: *n* (IQR)				
Left vs right vs bilateral	92.3 (86.1 – 99.0) vs 93.5 (87.5–99.9) vs 0	< 0.0001	86.5 (79.0–97.4) vs 87.6 (80.2–95.6) vs 0	< 0.0001
Protocol violation during primary surgery: *n* (IQR)				
Yes vs no	88.8 (81.2–97.0) vs 94.5 (88.6–100)	0.321	86.9 (79.6–94.9) vs 89.6 (81.9–97.9)	0.56
R0 status after primary surgery: *n* (IQR)				
Yes vs no	95.5 (90.6–100) vs 88.8 (81.2–97.0)	0.2	88.1 (80.7–96.3) vs 86.0 (77.5–93.3)	0.46
PRE: *n* (IQR)				
Yes vs no	89.5 (82.9–96.7) vs 96.1 (90.9–100)	0.19	85.0 (78.0–92.7) vs 88.9 (80.8–97.8)	0.43
Procotol violation during PRE: *n* (IQR)				
Yes vs no	97.3 (92.2–100) vs 92.3 (78.9–100)	0.36	85.5 (75.2–97.1) vs 92.3 (78.9–100)	0.59
R0 status after PRE: *n* (IQR)				
Yes vs no	97.6 (93.0–100) vs 81.8 (61.9–100)	0.02	89.4 (80.9–98.8) vs 81.8 (61.9–100)	0.33

*OS* overall survival; *EFS* event-free survival; *IRS* International Rhabdomyosarcoma Study Group stage; *PTRMS* paratesticular rhabdomyosarcoma; *CI* confidence interval; *IQR* interquartile range *CWS* Cooperative Weichteilsarkom Studiengruppe; *SoTiSaR* European Soft Tissue Sarcoma Registry; *PRE* pretreatment re-excision

Comparison of resection status after PRE for the patients with localized PTRMS showed significant differences in the OS rates (R1 vs R0 [81.8% vs 97.6%]; *p* = 0.018; Fig. S4). Because Cox regression analysis could not be performed, we examined the prevalence of potential confounding factors among the groups being compared using binary uni- and multivariable regression models, which showed no differences between the two groups regarding treatment protocol/era, histology, patient's age at the time of diagnosis, tumor size, side distribution, PV-PS, or regional LN status based on the imaging (Table S2). Those patients with R1 status after PRE (failed local control) were treated on an individual basis, with intensified chemotherapy ± radiation therapy, depending on recommendations from the multidisciplinary CWS study board.

## Discussion

By combining the data from two prospective CWS trials and the recent registry, we performed a detailed analysis of the quality of primary and revisional surgeries and their impact on EFS and OS in a large cohort of patients with either localized or metastatic PTRMS.

We observed a 41.9% prevalence of PVs during the primary surgery, which corresponds with the previously reported rates ranging from 30% to 41%.^20–22^ Relative to other pediatric solid tumors, with a reported PV rate of 15% for renal tumors, the prevalence of PV-PS in PTRMS is relevantly higher.^23^ This can be attributed to the fact that malignant para-testicular pathologies are often not anticipated preoperatively because the most common indications for surgical exploration are non-tumoral testicular or para-testicular conditions such as testicular torsion, hydrocele of the spermatic cord, epididymal cysts, or varicocele. Although painless scrotal swellings should raise suspicion of tumorous conditions, malignant testicular or para-testicular tumors also can present acutely, often when a trauma leads to the discovery of a swollen scrotum.

If orchiectomy is not performed, the rate of R1 resection is significantly higher, reaching 75% for the patients enrolled in our study. Fortunately, PRE with R0 resection can mitigate the consequences of inappropriately performed initial surgery while achieving excellent EFS and OS, as demonstrated in our patient cohort and also in other studies.^20,22^ However, the patients with localized PTRMS in whom R0 after PRE was not achieved had significantly worse unadjusted OS but not EFS than the patients with R0 resection (OS: 97.6% vs 81.8%; *p =* 0.018). Although we showed that patients among the R0-PRE and R1-PRE subgroups were comparable when traditional logistic regression models were used, it was not possible to verify this finding using time-to-event data. To the best of our knowledge, this association was demonstrated for the first time in the current study and should therefore be considered for further evaluation and validation in other trials. Furthermore, our study showed that the prevalence of protocol-deviating PRE surgeries was higher in non-university hospitals than in university hospitals (*p <* 0.001). This finding raises questions about the role of patient centralization in ensuring the best possible care, and its role in both oncologic and non-oncologic pediatric surgical care is intensively debated.^24–27^

In correspondence with the current literature,^5–7^ patient age of 10 years or younger and tumor size of 5 cm or smaller were confirmed as positive prognostic factors for EFS in the IRS I–III cohort. Another relevant factor affecting both EFS and OS is the presence of bilateral tumors, which had the highest mortality rate (100%) in our study. Notably, in both patients, the diagnosis of the contralateral side was delayed, leading to a failure in local surgical control.

In this homogeneous group of patients with fusion-negtive PTRMS, we observed a 11.5% prevalence of radiologically pathologic RPLN in the IRS I–III cohort and a 61.5% prevalence of LN metastases in the IRS IV cohort. These LN metastases occurred predominantly retroperitoneally and frequently were combined with metastatic lesions at other sites (92.3%), including pulmonary metastasis (46.1%) and skeletal metastasis (76.9%).

The patients with metastatic PTRMS were older than the patients with localized disease, confirming previous data.^28^ In contrast to the recommendations in the recently published consensus paper,^10^ these patients did not undergo LN sampling routinely if they were older than 10 years because primary surgical sampling of RPLN was not recommended in the past CWS protocols. However, when the patients underwent surgical LN exploration as part of an individual staging procedure, histologically positive LNs were found in 20% of the cases across the overall sampled cohort (both inguinal and retroperitoneal LNs). Specifically, in the subgroup of patients who underwent RPLN sampling, the rate of histologically positive LNs was 42.1%. Notably, all these RPLNs had been radiologically pathologic on the initial imaging. Seven of the eight patients with positive LNs were older than 10 years and had local tumors larger than 5 cm.

The relatively low incidence of histologically affected LN may be attributable to the fact that the majority of the sampled LNs in our patient cohort were inguinal. Although inguinal LNs were sampled during primary surgery before initiation of chemotherapy, they are rarely affected in the absence of tumor invasion into the scrotal skin. Furthermore, RPLN sampling, performed as part of an individualized treatment plan, was predominantly performed after induction chemotherapy, which theoretically could influence histologic LN involvement. Nevertheless, these outcomes partly aligned with the findings from other trials that have shown a low prevalence of microscopic involvement in LNs when CT or MRI imaging also was negative.^22,29^ However, other studies demonstrated contrasting findings, suggesting limited reliance on imaging, with the hazard of potential undertreatment due to findings that a substantial proportion of negative LNs on imaging were histologically positive.^30,31^

According to the recently published European guidelines for imaging in pediatric and adolescent rhabdomyosarcoma, positron emission tomography (PET)/CT or PET/MRI are the recommended methods for detecting locoregional and distant metastatic disease in RMS, with biopsy performed for any doubtful LN involvement.^32^ For patients with PTRMS, the current international diagnostic standard for retroperitoneal locoregional LN evaluation involves surgical staging for all patients 10 years old or older regardless of the morphologic (MRI and/or CT) findings on the initial imaging^10,20^ However, the role of functional investigations, such as ^15^F-labeled fluorodeoxyglucose (FDG) PET/CT or PET/MRI in PTRMS staging needs further evaluation in prospective multicenter trials. This could potentially influence the future diagnostic recommendations, with a shift from the invasive RPLND to non-invasive functional imaging.

One important finding of this analysis was that metastatic disease is very rare. Of 13 patients in the IRS IV cohort, 12 had regional and 8 had metastatic LN involvement, as determined by imaging, in combination with other metastatic regions, including lung and bone metastases, with an overall prevalence of combined metastatic disease in 92.3% of the patients.

The findings of our study should be considered in light of its limitations. First, the patients were enrolled in three different trials during a 27-year-long examination period with evolving protocols, potentially influencing the generalizability of our findings. Second, the quality assessment of surgical procedures was solely reliant on the operative notes, which may have been limited in capturing specific procedural details. Finally, due to the low number of events, it was not possible to conduct a Cox regression analysis. Such a “low-event multivariate model” would be compulsorily overfitted, resulting in nonsensical confidence intervals leaking statistical power.^33^

In conclusion, patients with localized PTRMS have excellent EFS and OS outcomes, which have remained consistent throughout the three prospective trials during the last 27 years. The quality of surgical local control has shown unsatisfactory results, with a high prevalence of PV-PS and a significant rate of initially incomplete resections. However, performance of PRE has demonstrated the potential to regain excellent EFS and OS. We found that R1 resection after PRE was linked to a decrease in unadjusted OS, suggesting its potential role as a prognostic surgical factor for patients with localized PTRMS.

### Supplementary Information

Below is the link to the electronic supplementary material.

Supplementary file1 (DOCX 725 kb)

## Data Availability

The data that support the findings of this study are available on request from the corresponding author.
