# Supplementary material for: Importance of Adequate Surgical Local Control in Fusion-Negative Para-Testicular Rhabdomyosarcoma: Data From the Cooperative Weichteilsarkom Studiengruppe Trials (CWS-96 and CWS-2002P) and the European Soft Tissue Sarcoma Registry (SoTiSaR)
Source: Ann Surg Oncol. 2024 Jun 15;31(9):6209–19. doi: 10.1245/s10434-024-15568-3 (PMC11300477; doi:10.1245/s10434-024-15568-3)
Supplement: Supplementary file 1 — Supplementary file1 (DOCX 725 kb) [file 10434_2024_15568_MOESM1_ESM.docx]

**Supplementary Table 1. Differences between different CWS trials regarding risk group stratification, chemotherapy, and radiotherapy**

|  | **Risk groups (IRS, pT, age, tumor size, nodal status, pathological subtype)** | **Chemotherapy** | **Radiotherapy** |
| --- | --- | --- | --- |
| **CWS-96** | Low: IRS I, pT1 | VA (20 wk) | Local tumor: only after incomplete resection  Lymph nodes without RPLND: eRMS with CR: no RT  eRMS with GR: 32 Gy  eRMS with PR: 44.8 Gy |
|  | Standard: IRS I, pT2 or IRS II, IRS III, N0 | I^2^VA (26 wk) |  |
|  | High: IRS I-III, N1 | VAIA or CEVAIE (randomization) |  |
| **CWS-2002P** | Low: IRS I, ≤ 10 y and ≤ 5 cm | VA (20 wk) |  |
|  | Standard: Standard IRS I, > 10 y or > 5 cm;  IRS II, II, age < 10 y, < 5cm, N0 | I^2^VA (25 wk) or I^2^VA-VA (25 wk, based on response) |  |
|  | High: IRS I-III, N1 | VAIA and E-CYC/VBL |  |
| **SoTiSar** | Low: IRS I, N0 | VA | Local tumor: only after incomplete resection  Lymph nodes without RPLND: eRMS with CR: no RT  eRMS with GR: 32 Gy  eRMS with PR: 44.8 Gy |
|  | Standard: IRS I-III, ≤ 10 y and ≤ 5 cm, N0 | VA and alkylating |  |
|  | High: eRMS, IRS II or IRS III, > 10 y or > 5 cm, N0 or N1 (any age and size); aRMS, IRS I-III, N0, |  |  |
|  | Very high: aRMS, IRS II-III, any age and size, N1. | VAIA |  |

Abbreviations: CWS, Cooperative Weichteilsarkom Studiengruppe; SoTiSaR, European Soft Tissue Sarcoma Registry; RPLND, retroperitoneal lymph node dissection; eRMS, embryonal rhabdomyosarcoma; aRMS alveolar rhabdomyosarcoma; CEVAIE Ifosfamide, Vincristine, Actinomycin-D, Carboplatin, Epirubicin; E-CYC/VBL, Cyclophosphamide, Vinblastin, Etoposide; EVAIA, Ifosfamide, Etoposid, Adriamycin, Actinomycin-D, Vincristine; I^2^ VA, Ifosfamide, Vincristine, Actinomycin-D; I^2^ VA-VA, Ifosfamide, Vincristine, Actinomycin-D; IRS, International rhabdomyosarcoma study group stage; VA, Vincristine, Actinomycin-D; VACA, Cyclophospamide, Adriamycin, Actinomycin-D; VAIA, Ifosfamide; Vincristine, Actinomycin-D, Adriamycin. CR: complete response; GR: good response; PR: partial response; RT: radiotherapy;

**Supplementary Table 2 Prevalence of potential confounding factors among patients with R0 and R1 status following PRE**

|  | **R0** | **R1** | ***P***  ***(univariate)*** | ***P***  ***(multivariate)*** |
| --- | --- | --- | --- | --- |
| **Treatment protocol/era**  CWS-96  2002P  SoTiSaR | 19 (86.4%)  19 (82.6%)  20 (83.3%) | 3 (13.6%)  4 (17.4%)  4 (16.7%) | 0.936 | 0.399 |
| **Histology**  eRMS  ssRMS  aRMS | 47 (83.9%)  10 (100%)  1 (33.3%) | 9 (16.1%)  0 (.0%)  2 (66.7%) | 0.022 | 0.095 |
| **Age, years**  ≤ 10  > 10 | 34 (82.9)  24 (85.7) | 7 (16.7)  4 (13.8) | 0.756 | 0.287 |
| **Tumor size, cm**  ≤ 5  > 5 | 36 (92.3%)  20 (71.4%) | 3 (7.7%)  8 (28.6%) | 0.042 | 0.077 |
| **Side distribution**  Left  right | 30 (90.9%)  28 (80.0%) | 3 (9.1%)  7 (20.0%) | 0.204 | 0.096 |
| **Protocol violation during initial surgery**  Yes  No | 41 (85.4%)  13 (76.5%) | 7 (14.6%)  4 (23.5%) | 0.398 | 0.638 |
| **Radiologically pathological LN**  Yes  No | 3 (100%)  12 (70.6%) | 0 (0.0%)  5 (29.4%) | 0.278 | Due to low number of LN evaluated patients no multivariate analysis performed |

Abbreviations: CWS, Cooperative Weichteilsarkom Studiengruppe; SoTiSaR, European Soft Tissue Sarcoma Registry; PRE, pretreatment re-excision;

**Supplemental Figure 1. Age distribution in IRS I-III and IRS IV cohorts**

**
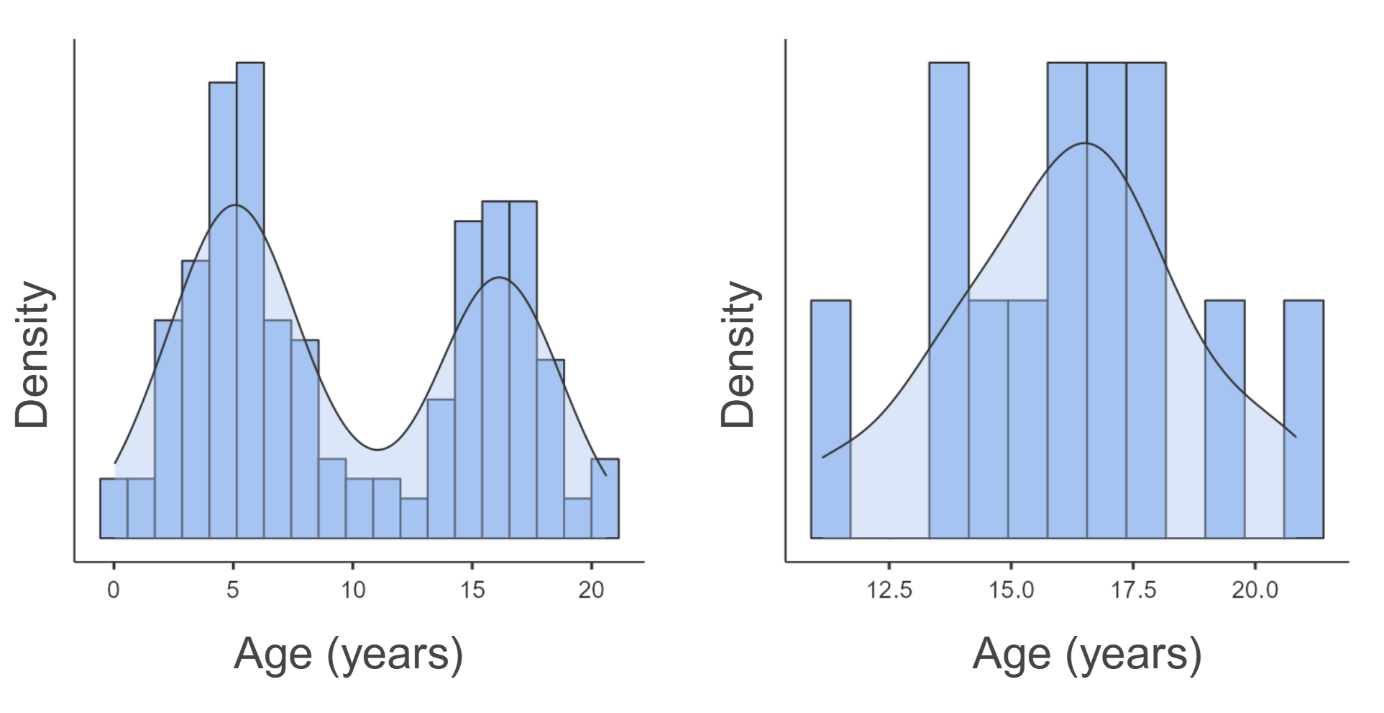
**

Abbreviations: IRS, International rhabdomyosarcoma study group stage;

**Supplementary Figure 2. Flowchart of primary surgery within the IRS I-III cohort**

**
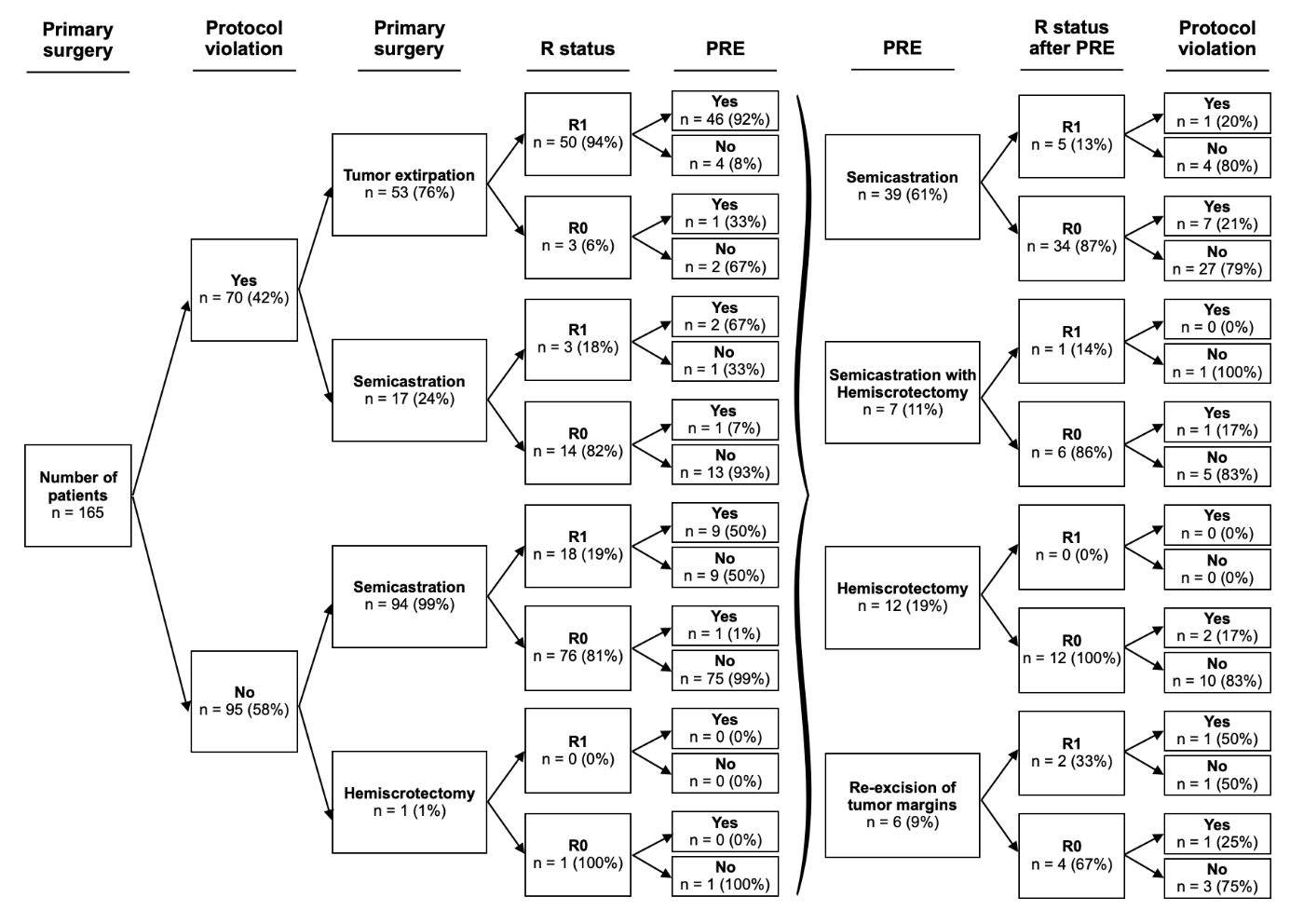
**

Abbreviations: IRS, International rhabdomyosarcoma study group stage; PRE, pretreatment re-excision; The total number of cases (n=165) represents those included in the creation of the variable tree, with exclusions made for any cases with missing information; The difference in the absolute number of patients who underwent PRE (n=60 before the right brace and n=64 after the right brace) results from the nature of the variable tree, where only cases with complete variable information are included.

**Supplementary Figure 3. Lymph node biopsy in IRS I-III cohort**

**
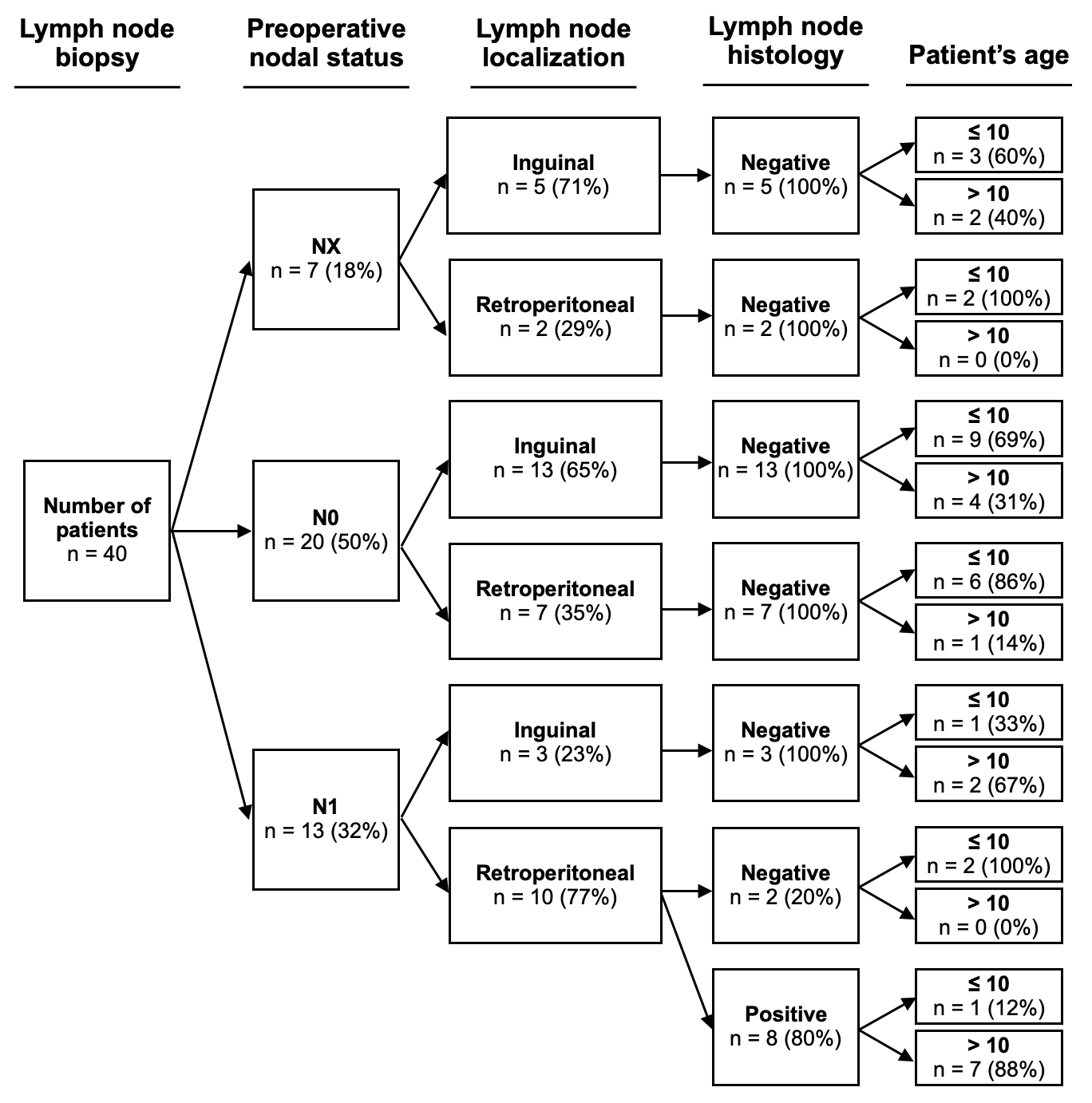
**

Abbreviations: IRS, International rhabdomyosarcoma study group stage;

**Supplementary Figure 4. 5-year OS analysis in IRS I-III and by R status after primary re-excision surgery**

**
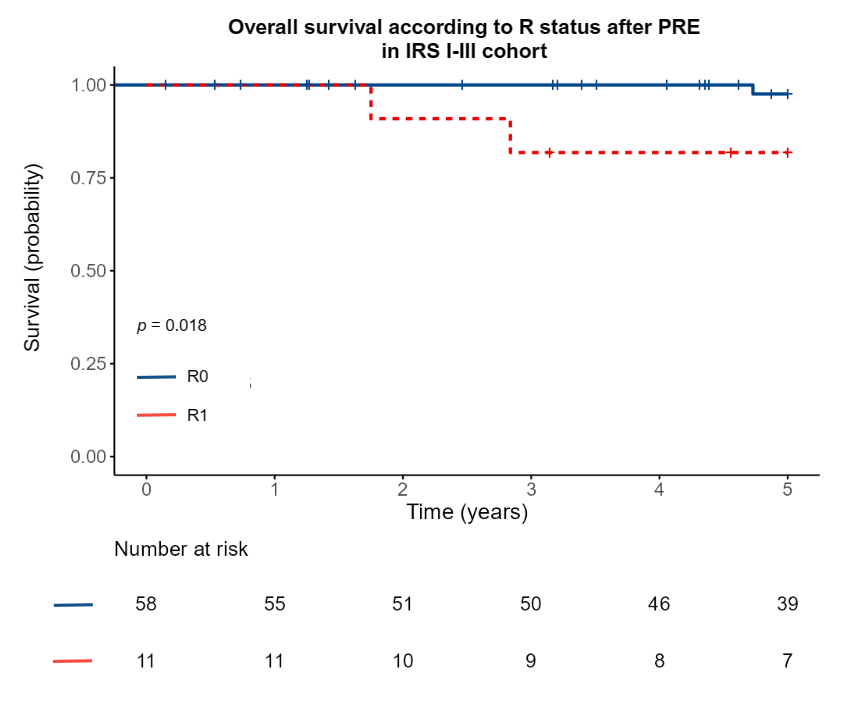
**

Abbreviations: IRS, International rhabdomyosarcoma study group stage; OS, overall survival; PRE primary re-excision surgery;
